# Nitroimidazole - albendazole combination therapy, quinacrine use, and risk factors in nitroimidazole-refractory giardiasis: a prospective multicentre observational study

**DOI:** 10.1186/s12879-026-13833-2

**Published:** 2026-06-20

**Authors:** Kristine Mørch, Peter Chiodini, Laura Nabarro, Raisa Hannula, Andreas Neumayr, Christel Gill Haanshuus, Christina Saghaug, Bjørn Blomberg, Frank Olav Dahler Pettersen, Helene Heitmann Sandnes, Kurt Hanevik, Nina Langeland

**Affiliations:** 1 National Centre for Tropical Infectious Diseases, Department of Medicine, Haukeland University Hospital, PO Box 1400, Helse Bergen HF, N-5021 Norway; 2 Department of Clinical Science, University of Bergen, Bergen, Norway; 3 Department of Clinical Parasitology, Hospital for Tropical Diseases, University College London Hospitals NHS Foundation Trust, London, UK; 4 London School of Hygiene and Tropical Medicine, London, UK; 5 Department of Infectious Diseases, St. Olav’s Hospital, Trondheim University Hospital, Trondheim, Norway; 6 Department of Medicine, Swiss Tropical and Public Health Institute, Basel, Switzerland; 7 University of Basel, Basel, Switzerland; 8 College of Medicine and Dentistry, Division of Tropical Health and Medicine, James Cook University, Townsville, Australia; 9 Regional Advisory Unit for Imported and Tropical Diseases, Department of Infectious Diseases, Oslo University Hospital, Ullevål, Oslo, Norway; 10 Norwegian Institute of Public Health, Oslo, Norway

**Keywords:** *Giardia*, Refractory, Second line, Treatment, Assemblage, Metronidazole, Tinidazole, Ornidazole, Albendazole, Quinacrine

## Abstract

**Background:**

Nitroimidazole-refractory giardiasis is an increasing problem. We present data on efficacy of a treatment ladder and on clinical characteristics and assemblage types in nitroimidazole-refractory giardiasis.

**Methods:**

We conducted a prospective clinical observational study of adult patients with giardiasis at four centres in Norway and England during 2009 – 2024. Patients with nitroimidazole-refractory giardiasis were treated with albendazole plus a 5-nitroimidazole followed by quinacrine (mepacrine) if failure. Treatment efficacy was defined as negative stool microscopy and/or PCR four to six weeks after treatment. For analyses of assemblage types and risk factors for treatment failure, patients from a previously published Swiss treatment study were additionally included. Assemblage typing of *Giardia* isolates was performed collectively in the same laboratory by real-time PCR targeting the glutamate dehydrogenase gene (gdh). Predictors identified by univariate analyses were analysed by multivariate logistic regression for association with nitroimidazole failure.

**Results:**

A total of 120 patients were prospectively included; 59 of these had nitroimidazole refractory giardiasis and were treated according to the treatment ladder. In addition, 20 patients from the Swiss cohort were included for assemblage and risk factor analyses. A repeated course of nitroimidazole cured only 24% (5/21). Metronidazole or tinidazole in combination with albendazole cured 76% (35/46). Quinacrine was effective in 100% (15/15). Assemblage B was more common in travellers from India and Africa, but only acquisition of infection in India (aOR 11.9; 95%CI 2.94, 47.6) and more recent year of diagnosis (aOR 1.18, 95% CI 1.03, 1.35) were associated with nitroimidazole failure in multivariate analysis.

**Conclusion:**

Second line treatment with nitroimidazole in combination with albendazole, and third line treatment with quinacrine, are effective options in nitroimidazole-refractory giardiasis. Nitroimidazole failure seems to be highly associated with infection acquired in India, but not with assemblage A or B. Further studies of resistance mechanisms are needed.

**Supplementary Information:**

The online version contains supplementary material available at https://doi.org/10.1186/s12879-026-13833-2.

## Background

Giardiasis is a protozoan intestinal infection with clinical manifestations ranging from asymptomatic self-limiting infection to severe diarrhoea with weight loss and malabsorption [1–3]. It causes malnutrition and stunting due to repeated infections in low- and middle-income countries, while in high-income countries giardiasis present as traveller’s diarrhoea and epidemics during waterborne outbreaks [4–6].

First line treatment with a 5-nitroimidazole, either metronidazole for 5–7 days or the long acting nitroimidazoles tinidazole, secnidazole or ornidazole single dose, has previously had high efficacy in controlled clinical studies [7]. However, clinicians now observe increasing nitroimidazole treatment failure. Cure rate after metronidazole was as low as 54% (248/456) in a recent prospective study from Cuba [8]. Several retrospective studies report particularly high failure rate among travellers to Asia: A study from England found an increase of nitroimidazole failure from 15% (8/53) in 2008 to 40% (35/87) in 2013, and 50% (51/101) among travellers to India [9]. A study from Spain in 2007–2009 found nitroimidazole failure in 22% (21/95) overall and in 33% (12/36) among travellers to Asia [10]. A population-based study from Sweden (*n* = 4285) found nitroimidazole failure increasing from 2% to 3% totally, and from 9% to 17% in travellers to India, during the period 2008 to 2020 [11]. And a study from Germany reported 30% (93/308) first-line treatment failure between 2007 and 2016, and 49% (62/126) among travellers to India [12].

Only a few studies have investigated treatment efficacy in nitroimidazole-refractory giardiasis [7]. Quinacrine (mepacrine) is effective in close to 100% of cases [13, 14], also in immunocompromised patients [15], but is used with caution due to rare but potential severe neuropsychiatric side effects. Combination therapy seems to be more effective than monotherapy with a different class of drug or repeated courses with increased dose/duration with the same drug [7]. A benzimidazole (albendazole or mebendazole) in combination with a 5-nitroimidazole, was effective in 79% (30/38), 87% (100/115) and 82% (9/11) of metronidazole-refractory cases in three prospective single-arm cohort studies from Norway and Cuba respectively [8, 16, 17].

*Giardia* can be diagnosed by microscopic detection of cysts in stool concentrates, by antigen tests or by polymerase chain reaction (PCR). However, the choice of drugs in treatment failure is empirical because in vitro antimicrobial susceptibility testing is hampered by the parasite’s limited ability to grow in culture and by different growth rates of *Giardia* isolates [18, 19]. Molecular resistance markers are being investigated but are not yet identified [20–24].

The zoonotic protozoan *Giardia duodenalis* is grouped into assemblages A-H, with A and B being the most common in humans [25, 26]. Assemblages A and B have different genetic characteristics and geographical distribution, but up to now studies have not reported differences in clinical disease [27–30].

The main objective in this study was to evaluate the efficacy of metronidazole or tinidazole in combination with albendazole as second line treatment, and quinacrine as third line treatment, in nitroimidazole-refractory giardiasis.

Secondary objectives were to describe clinical disease, origin of acquisition of infection, and assemblage type distribution, and evaluate the association with nitroimidazole-refractory *Giardia* infection.

## Methods

### Patient selection

Adult patients were prospectively included in an observational single-arm cohort study at university hospitals in Bergen, Trondheim and Oslo in Norway and London, England in the period 2016–2024, and in Bergen additionally in the period 2009–2014. All the patients included prospectively from London in this study were also included in a larger retrospective audit on the epidemiology and management of giardiasis between 2020 and 2024 published in 2026 [31]. In Bergen and London, patients were included both from *Giardia* positive treatment naive patients diagnosed at the routine microbiology laboratory and from patients referred for treatment refractory giardiasis, while in Trondheim and Oslo patients were included from referred treatment refractory cases only. In London, patients with immunodeficiency were excluded. All patients were treated according to a predefined treatment ladder. Clinical information and travel history were collected on a predefined form.

Stool samples and clinical and demographic data were included from a Swiss cohort included in a previously published European prospective multicentre study (2014–2020) evaluating combination treatment with albendazole plus chloroquine versus quinacrine in nitroimidazole-refractory giardiasis (*N* = 20) [32]. The samples and data from Switzerland were additionally included, as assemblage and risk factor analyses had not previously been performed on this dataset. *Giardia* positive stool samples from all centres were collected and analysed at Haukeland University hospital for assemblage types.

### Diagnosis and evaluation of treatment efficacy

*Giardia* was diagnosed in routine microbiology laboratories with microscopy of cysts in stool and/or by PCR. The same method was applied before and after treatment. *Giardia* specific PCR or multiplex stool PCR panel was used according to the routines at the laboratories. Treatment efficacy was defined as negative microscopy and/or PCR four to six weeks after treatment, or clinical cure in cases were follow up parasitological tests were not performed. Clinical symptoms including adverse events were evaluated by the treating physician at the outpatient clinic on all patients at baseline and follow up visits.

### Treatment ladder design

Patients from Norway and England were treated according to the following treatment ladder:

- First line: Metronidazole 400 mg or 500 mg three times daily (TID) for five days, or tinidazole 2 g single dose (SD)
- Second line: Albendazole 400 mg two times daily (BID) + metronidazole 400 mg or 500 mg BID for seven days, or albendazole 400 mg BID for seven days + tinidazole 2 g SD day one and 2 g SD day seven
- Third line: Quinacrine 100 mg TID for seven days

Other established anti-*Giardia* drugs or repeated courses of nitroimidazole were given at the discretion of the treating physician as shown under results.

Patients from Switzerland were treated with ornidazole first line and quinacrine 100 mg TID for five days second line in refractory cases as previously described [32].

### Assemblage typing

Assemblage typing of *Giardia* isolates was performed collectively at Haukeland University hospital in Bergen, Norway. To obtain high quality *Giardia* DNA for assemblage typing, clinical samples from Norway and England (*N* = 81) were processed by first isolating *Giardia* cysts from fresh stool using immunomagnetic separation with Dynabeads G-C combo kit (Life Technologies, Carlsbad, CA, USA) following the manufacturer’s procedure and method as described in [24] and [22]. Samples were freeze-thawed twice before extracting DNA applying a MagAttract HMW DNA kit (Qiagen, Hilden, Germany). For samples collected early in the study from Norway (*N* = 29), and the samples collected in Switzerland (*N* = 25), the DNA was extracted directly from fresh stool applying QIAamp DNA Stool Mini Kit (Qiagen), or from frozen stool applying QIAamp PowerFecal Pro DNA kit (Qiagen).

The isolates were analysed for assemblage type by applying a real-time PCR targeting the glutamate dehydrogenase gene (*gdh*), using previously published primers [33], with a few modifications (lowering the melting temperatures); forward AII 5’ GGCAAGTCCGACAACGA 3’, forward B 5’ GGCAAGTCGGACAACGA 3’, and reverse AII and B 5’ GCACATCTCCTCCAGGAAGTAG 3’. At the start of the study, the probe 6FAM-AGCTCCAG+AGGCACG+TCGG-BHQ-1, was used to detect *Giardia* DNA, followed by sequencing of the *gdh* PCR-product (261 bp) to determine the assemblage type. The oligos were provided by Eurogentec (Seraing, Belgium). Later in the study, the assemblage typing was simplified by using SYBR Green followed by melting curve analysis. Amplification parameters and reaction details for both assays are given in Supplement Table 1.

### Statisticial methods

In descriptive analysis, dichotomous variables were given as proportions and continuous variables as medians with interquartile range (IQR). Univariable and multivariable analyses of risk factors for nitromididazole failure and association with assemblage A or B included demographic variables, year of diagnosis, origin of infection, symptoms, duration of disease and outcome. Missing data were handled using complete case analysis; observations with missing values were excluded from relevant analyses. No imputation was performed. In univariable analyses we calculated unadjusted odds ratio (OR), 95% confidence interval (CI) and *p*-values, using logistic regression for both dichotomous variables and continuous explanatory factors. Age, gender and significant risk factors with *p* < 0.05 in the univariable analyses were included in multivariable analyses. For multivariable analysis, we used logistic regression and presented results as adjusted OR (aOR) and 95% CI. Statistical analyses were performed using SPSS Statistics version 26 (IBM, Armonk, NY, USA).

## Results

A total of 120 patients were prospectively included in Norway and England; 59 of these had nitroimidazole refractory giardiasis. In addition, 10 nitroimidazole responsive and 10 nitroimidazole refractory patients from the Swiss cohort were included for assemblage and risk factor analyses. Among all, median age was 41 years (range 18 to 85 years) and 51% were female. Characteristics and clinical findings are shown in Table 1.

**Table 1:** Demographics, baseline characteristics and clinical outcome of patients with giardiasis (*n* = 140)

	Patients
	n/total^1^(%)
**Age**, years; median (IQR), *n* = 140	41 (29–57)
**Gender**	
Male	68/140 (49)
Female	72/140 (51)
**Study centre**	
Bergen, Norway	68/140 (49)
Trondheim, Norway	9/140 (6)
Oslo, Norway	1/140 (1)
London, England	42/140 (30)
Basel, Switzerland	20/140 (14)
**Giardiasis previously**	5/108 (5)
**Immunodeficiency**^**2**^	5/91 (6)
**Pets in the household**	6/52 (12)
**Year diagnosed**; median (IQR) *n* = 140	2018 (2015–2023)
**Place of *Giardia* acquisition**	
India	38/131 (29)
Asia other^3^	11/131 (8)
Africa^4^	36/131 (28)
Europe^5^	29/131 (22)
Latin America^6^	17/131 (13)
**Symptoms**	
Diarrhoea	101/114 (89)
Diarrhoea n/day; median (IQR) *n* = 80	5 (3–9)
Abdominal pain	37/43 (86)
Foul flatulence	82/113 (73)
Lack of appetite	22/30 (73)
Bloating	62/92 (67)
Weight loss	59/97 (61)
Weight loss, kg; median (IQR) *n* = 91	3 (0–7)
Nausea	53/88 (60)
Vomiting	17/40 (43)
Fever	11/34 (32)
No symptoms	2/140 (1)
***Giardia* assemblage**	
Assemblage B	67/100 (67)
Assemblage A	33/100 (33)
**Duration of symptoms,** median (IQR)	
Weeks until diagnosis, *n* = 108	4 (2–8)
Weeks until cure, *n* = 105	9 (4–20)
**Coinfections**	
*Ascaris*	1
Taeniasis	1
*Campylobacter*	2
*Clostridioides difficile*	3
*Salmonella paratyphi*	1
*Shigella*	1
Norovirus	1
Malaria	3
HIV	1
**Nitroimidazole treatment group**	
Nitroimidazole refractory	64/137 (47)
Nitroimidazole responsive	73/137 (53)
**Clinical outcome**	
Complete recovery	95/127 (75)
Improvement	18/127 (14)
Persistent symptoms	11/127 (9)
Death	3/127 (2)

^1^Discrepancies from total (*n* = 140) due to missing values. Data from Basel, Switzerland only available for age, gender, year diagnosed, place of *Giardia* acquisition, *Giardia* assemblage, nitroimidazole treatment group and clinical outcome

^2^Kidney transplant/Sandimmune (*n* = 1), methotrexate (*n* = 1), IgA deficiency (*n* = 1), renal failure (*n* = 2)

^3^Bangladesh, Cambodia, Georgia, India or Bhutan, India or China or Turkey, India or Malaysia (*n* = 2), Indonesia, Malaysia or Indonesia, Sri Lanka, Pakistan

^4^Cape Verde, Egypt (*n* = 2), Ghana (*n* = 4), Guinea, Ivory Coast (*n* = 3), Kenya (*n* = 4), Tanzania (*n* = 2), Zanzibar (*n* = 7), Kenya or Zanzibar, Madagascar (*n* = 2), Malawi, Marocco, Sudan (*n* = 2), Botswana or South Africa, Togo, Uganda (*n* = 2), Zambia or Ghana

^5^England (*n* = 5), England or Switzerland, Norway (*n* = 6), Norway or Italy, Norway or Germany, Romania or Moldova or Spain, Spain (*n* = 2), Turkey, Turkey or Greece, Denmark

^6^Brazil (*n* = 3), Bolivia or Brazil, Colombia (*n* = 3), Colombia or Panama, Cuba (*n* = 3), Dominican Republic, Ecuador, Mexico, Nicaragua (*n* = 3), SA undefined

**Fig. 1:**
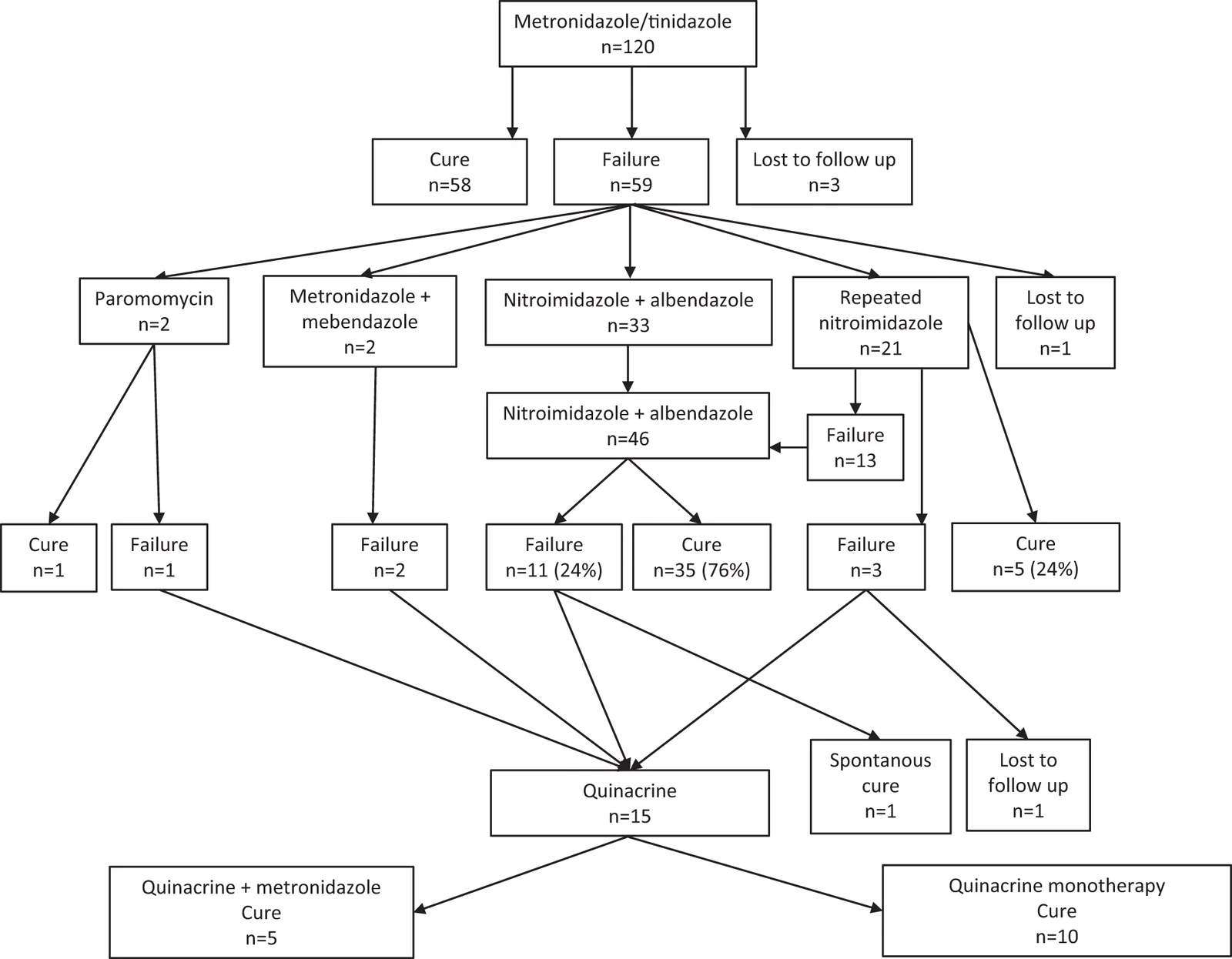
Results of *Giardia* treatment in patients with nitroimidazole failure

Among the 120 patients managed according to the treatment ladder, 95 received metronidazole and 25 tinidazole; 45 and 14 patients, respectively, were nitroimidazole refractory. Second-line treatment with nitroimidazole in combination with albendazole was administered to 46 patients. Other regimens, prescribed at the discretion of the treating physician, included repeated courses of nitroimidazole, paromomycin, or metronidazole combined with mebendazole (Fig. 1).

Out of the 46 patients treated with metronidazole or tinidazole in combination with albendazole, 76% (35/46) responded (Fig. 1). The cure rate of this combination was 13/22 (59%) among patients included in Norway, and 22/24 (92%) among patients included in England. Among patients who had received a repeated course of nitroimidazole, only 24% (5/21) were cured (Fig. 1). One was cured by tinidazole after metronidazole and four were cured by repeated metronidazole. Seven failed on tinidazole after metronidazole, two failed on repeated tinidazole and seven failed on repeated metronidazole courses. All treatment refractory cases were cured by quinacrine (*n* = 15). Ten were cured by quinacrine monotherapy for one week and four by quinacrine in combination with metronidazole for one week. One patient had repeated relapses and improved after various regimens, including repeated courses of metronidazole, quinacrine for one week and quinacrine in combination with metronidazole for two weeks. Two patients had headache, nausea and dizziness when treated with metronidazole and tinidazole, and one patient developed a rash from albendazole, while the rest of patients treated and all patients who received quinacrine completed the course with no reported severe side effects. Duration of symptoms before effective cure was median 15 weeks (IQR 10–24) in refractory cases compared to 4 weeks (IQR 2–8) in responsive cases.

Fourteen patients had coinfections (Table 1). Three patients had *Clostridioides difficile* and were treated with metronidazole, one patient had well-controlled HIV, and other patients received treatment for bacterial or parasitic infections with agents not presumed to have anti-*Giardia* activity.

Three patients aged 83–85 years died from conditions considered unrelated to *Giardia* infection. One was hospitalised with ileus and died due to colon cancer and pulmonary embolism. One was multimorbid with chronic kidney disease and died from perforated appendicitis, after three years of chronic giardiasis with diarrhoea refractory to repeated courses of metronidazole. One patient who was hospitalised with diarrhoea, end stage renal failure and coinfection with *Giardia* and *Clostridioides difficile* responded to metronidazole (as proven by two negative *Giardia* PCR tests) but did not show improvement of diarrhoea.

*Giardia* was diagnosed by microscopy in 28% (33/120), by PCR in 33% (39/120), by microscopy + PCR in 38% (46/120) and by duodenal biopsy in 2% (2/120) of the patients. Treatment response was identified by a negative parasitological test in 104 patients (by microscopy + PCR in 54, only PCR in 41, only microscopy in 4, and microscopy or PCR undefined in 5 patients), only clinically in 13 patients, and three were lost to follow up. Among the patients who had a negative parasitological test at follow up, 63% (65/104) experienced complete recovery, 16% (17/104) had improved, 11% (11/104) had persistent symptoms, one died, and clinical outcome data were missing for 10 patients. Among those who were PCR negative at follow up, 70% (62/89) had complete recovery, 19% (17/89) had improved and 10% (9/89) had persistent symptoms. Among those negative by microscopy or by PCR/microscopy undefined, three had complete recovery and one had persistent symptoms. Among 13 patients who had no microbiological test at follow up, 10 had complete recovery, one had improved and two died of reasons considered unrelated to giardiasis.

Table 2 shows factors associated with nitroimidazole failure in patients from all the sites (*n* = 140).

**Table 2:** Characteristics and findings associated with nitroimidazole failure

Characteristics	Overall, n/N	Nitroimidazole refractory N = 64	Nitroimidazole responsive N = 73	Univariable	Multivariable
				OR (95% CI)	aOR (95% CI)
**Age** years; median (IQR)	137	42 (29–57)	38 (31–60)	1.00 (0.98, 1.02)	1.01 (0.98, 1.04)
**Gender**; male	68/137	58% (37/64)	42% (31/73)	1.86 (0.94, 3.66)	2.14 (0.69, 6.58)
**Weeks** until diagnosis; median (IQR)	106	4 (2–8)	4 (2–8)	1.00 (0.97, 1.02)	
**Immunodeficiency**	5/91	4% (2/48)	7% (3/43)	0.58 (0.09–3.65)	
**Pets in household**	6/52	17% (5/30)	5% (1/22)	4.20 (0.45–38.83)	
**Year diagnosed**; median (IQR)	137	2019 (2017–23)	2017 (2017–23)	**1.10 (1.01–1.20)**	**1.18 (1.03, 1.35)**
**Place of infection**					
India	38/129	50% (30/60)	12% (8/69)	**10.4 (3.50, 31.3)**	**11.95 (2.94, 47.6)**
Asia other	11/129	8% (5/60)	9% (6/69)	2.31 (0.56, 9.52)	1.71 (0.24, 12.5)
Europe	29/129	22% (13/60)	23% (16/69)	2.26 (0.78, 6.49)	2.90 (0.63, 13.3)
Latin America	17/129	5% (3/60)	20% (14/69)	0.59 (0.14, 2.56)	0.81 (0.10, 6.45)
Africa	34/129	15% (9/60)	36% (25/69)	1.00 (ref.)	
***Giardia* assemblage**					
Assemblage B	66/99	77% (37/48)	57% (29/51)	**2.55 (1.07, 6.10)**	1.85 (0.55, 6.21)
Assemblage A	33/99	23% (11/48)	43% (22/51)	1.00 (ref.)	
**Symptoms**					
Diarrhoea	100/113	89% (46/52)	89% (54/61)	0.99 (0.31, 3.17)	
Bloating	62/92	74% (37/50)	60% (25/42)	1.94 (0.80, 4.68)	
Abdominal pain	36/41	83% (10/12)	90% (26/29)	0.58 (0.08, 3.98)	
Weight loss	58/96	60% (30/50)	61% (28/46)	1.04 (0.46, 2.35)	
Nausea or vomiting	56/92	50% (21/42)	70% (35/50)	0.43 (0.18, 1.01)	
Foul flatulence	80/111	74% (39/53)	71% (41/58)	1.16 (0.50, 2.66)	
**Clinical outcome**					
Complete recovery	95/127	68% (41/60)	81% (54/67)	1.00 (ref.)	
Improvement	18/127	18% (11/60)	10% (7/67)	2.07 (0.73, 5.81)	
Persistent symptoms	11/127	10% (6/60)	8% (5/67)	1.58 (0.45, 5.56)	
Death	3/127	3% (2/60)	2% (1/67)	2.63 (0.23, 30.3)	

Abbreviations: OR, odds ratio; aOR, adjusted odds ratio; CI, confidence interval; IQR, interquartile range

Binary logistic regression for dichotomous and continuous variables in univariable and multivariable analyses

**Fig. 2:**
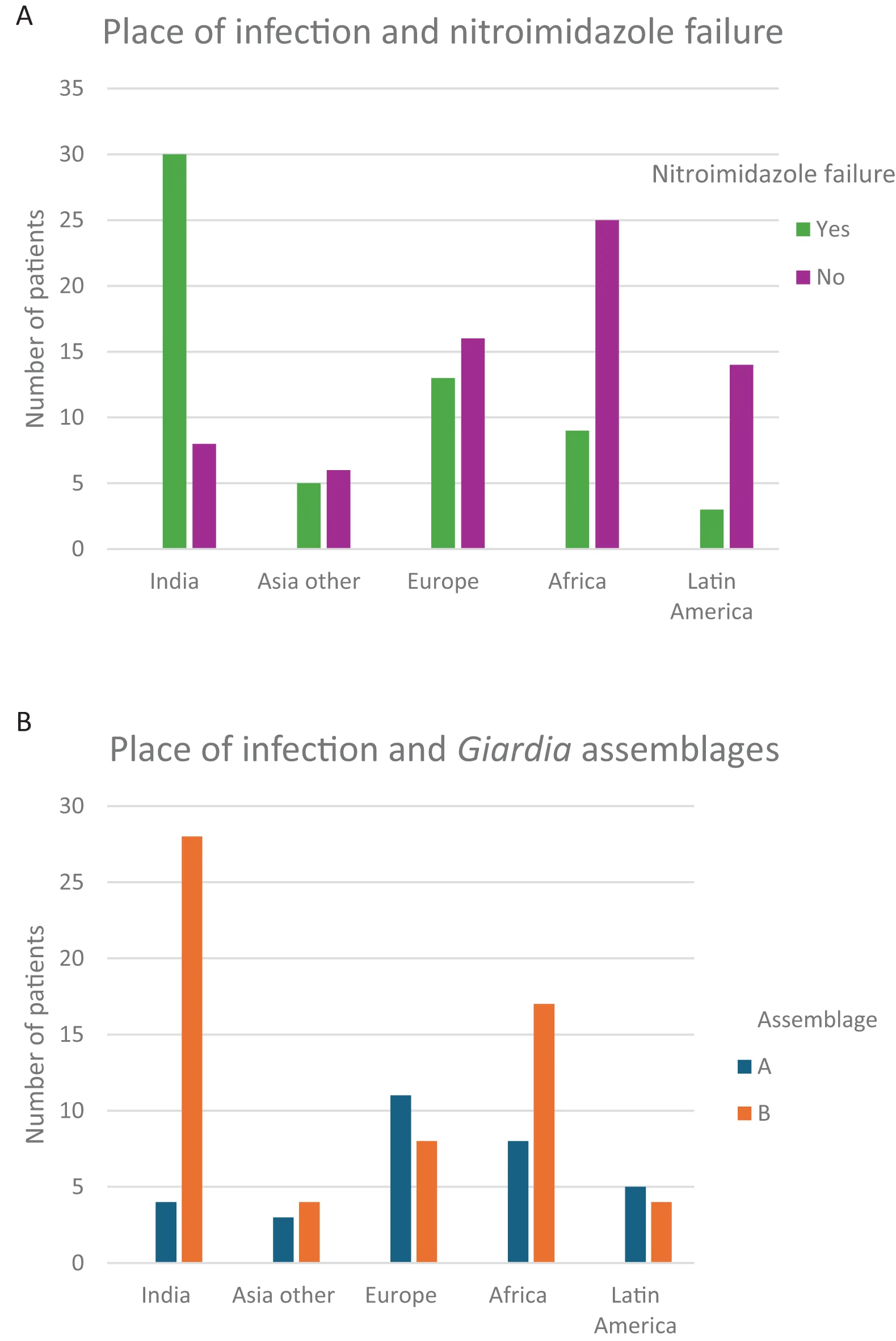
Place of infection associated with nitroimidazole failure (**A**) and with *Giardia* assemblages (**B**)

As many as 79% (30/38) of patients infected in India failed on nitroimidazole treatment (Table 2, Fig. 2).

In univariable analyses nitroimidazole failure was significantly associated with infections acquired in India (OR 10.4, 95% CI 3.50, 31.3), with a more recent year of diagnosis (median year 2019 compared to 2017) (OR 1.10, 95% CI 1.01, 1.20) and assemblage B (OR 2.55, 95% CI 1.07, 6.10). In the multivariable analysis, only more recent year of diagnosis (aOR 1.18, 95% CI 1.03, 1.35) and infection acquired in India (aOR 11.95; 95%CI 2.94–47.6) were significantly associated with nitroimidazole failure. There was a borderline, but not significant interaction term between India as origin of infection and assemblage B (estimate 2.23, p 0.146). In analysis restricted to participants infected with assemblage B, infection acquired in India remained a significant risk factor for nitroimidazole failure in the univariable model (OR 27.8, 95% CI 5.46, 142.85) (Table 3).

**Table 3:** Risk of nitroimidazole failure in patients with assemblage B (**a**), and in patients who acquired infection in India (**b**)

	Assemblage B (a)		OR (95% CI)^1^	India (b)		OR (95% CI)^1^
	Nitroimidazole refractory N = 35	Nitroimidazole responsive N = 26		Nitroimidazole refractory N = 26	Nitroimidazole responsive N = 6	
**Place of infection (a)**				-	-	
India	67% (24/35)	15% (47/26)	**27.8 (5.46, 142.9)**	-	-	
Asia other	6% (2/35)	8% (2/26)	4.67 (0.45, 47.6)	-	-	
Europe	11% (4/35)	15% (4/26)	4.67 (0.72, 30.3)	-	-	
Latin America	6% (2/35)	8% (2/26)	4.67 (0.45, 47.6)	-	-	
Africa	9% (3/35)	54% (14/26)	1.00 (ref.)	-	-	
**Assemblage B (b)**	-	-		92% (24/26)	67% (4/6)	6.00 (0.65, 55.56)

^1^Binary logistic regression, univariable analysis. Analysis restricted to participants with assemblage B (a) and analysis restricted to those who acquired infection in India (b)

In analysis restricted to those who acquired infection in India, there was no significant association between infection with assemblage B and nitroimidazole failure (OR 6.00, 95% CI 0.65, 55.56) (Table 3).

Asia including India was the most common place of acquiring infection (49/131, 37%), followed by Africa (36/131, 28%), Europe (29/131, 22%) and Latin America (17/131, 13%). *Giardia* infections in patients in Norway, England and Switzerland were acquired in India in 16, 46 and 40%, in Europe in 33, 15 and 0%, in Latin America in 13, 10 and 20% and in Africa in 30, 20 and 35% respectively.

*Giardia* assemblage B was found in 67% (67/100) and assemblage A in 33% (33/100). Assemblage B was found in 56, 79 and 85% of patients included in Norway, England and Switzerland respectively.

Assemblage B was more common after travel to India and Africa, while assemblage A was more common from Europe and Latin America (Fig. 2).

Younger age (median 38 compared to median 53 years, aOR 0.97, 95% CI 0.94, 0.99), male gender (57%, aOR 3.44, 95% CI 1.08, 10.9) and origin of *Giardia* acquisition were significant factors associated with genotype B. We found no significant association between assemblages and nitroimidazole failure in the adjusted analysis (aOR 0.53, 95% CI 0.17, 1.67). Clinical symptoms and year of diagnosis were not associated with assemblages in the univariable analyses.

## Discussion

### Treatment efficacy in nitroimidazole-refractory patients

In this study of nitroimidazole-refractory giardiasis, we found a high degree of efficacy of second line treatment with nitroimidazole in combination with albendazole (Fig. 1). Among patients who failed one or repeated courses of nitroimidazole, 76% (35/46) responded to nitroimidazole in combination with albendazole. This supports the efficacy shown previously. Nitroimidazole and albendazole showed synergistic effect in a small Italian controlled study [34], and 79% efficacy in a prospective observational study during a large outbreak in Norway [16]. In addition to these prospective studies, case series and retrospective studies have reported between 50 and 82% efficacy of this second line combination treatment [35]. Nitroimidazole in combination with mebendazole, a benzimidazole in the same class as albendazole, was effective as second line treatment in a prospective study from Cuba [8].

A repeated course of nitroimidazole monotherapy failed in 76% (16/21) and supports previous studies that this is not a good strategy compared to combination treatment for patients with treatment failure after nitroimidazole [35].

All treatment refractory cases were cured by quinacrine monotherapy or in combination with metronidazole (Fig. 1). The high efficacy of this drug is well described for treatment refractory giardiasis [7, 15, 35], but often recommended as third line treatment due to risk of rare but severe neuropsychiatric side effects, as described previously in one patient in the cohort from Switzerland in our study [32]. Quinacrine was a drug of choice for many years after it had first shown anti-*Giardia* efficacy in the 1930s [36], but today quinacrine is not readily available and must be prescribed under exemption from marketing authorisation in most countries. Although some new candidates such as auranofin and fumagillin are under investigation [37], giardiasis is a neglected disease, and the number of available treatment alternatives have been the same for decades. Given the increasing prevalence of nitroimidazole resistance, there is a need for improved access to quinacrine, as well as for continued research into new therapeutic agents.

### Characteristics of treatment refractory giardiasis

As much as 79% (30/38) of patients infected in India had failed on nitroimidazole treatment (Table 2, Fig. 2), compared to 47% (64/137) among total patients. Assemblage B was found in 88% of the cases from India and 68% from Africa, while assemblage A was predominant from Europe and Latin America (Fig. 2), in line with previous reports of assemblage distribution [30]. However, in the present study, assemblage type was not a significant risk factor for nitroimidazole failure in the adjusted analysis, where only acquisition of infection in India and recent year of infection remained as significant risk factors. This suggests that resistance mechanisms independent of assemblage genotype have developed in India where selection pressure is high due to widespread use of antibiotics and high prevalence of *Giardia* infection.

Nitroimidazole failure in travellers has increased over the years and is now reported in up to 50% [8, 9]. In this study we found the same trend, where the median year of diagnosis for nitroimidazole responsive cases was 2017 compared to 2019 for refractory cases (Table 2).

Duration of symptoms before *Giardia* diagnosis was similar in refractory and responsive cases, but the duration of symptoms before cure was higher (median 15 weeks) in refractory cases, reflecting the repeated courses of treatment and delay in clearing the infection. We have previously shown that duration of infection is a risk factor for post-infectious fatigue and irritable bowel syndrome in giardiasis [38, 39], which calls for rapid effective second line treatment also to reduce the risk of complications.

Subclinical infection is common in giardiasis, reported in up to 50% in experimental studies [2, 40]. Our study mainly included symptomatic patients due to the selection of patients in specialist clinics, and the reported symptoms were characteristic for giardiasis with diarrhoea, abdominal pain, foul flatulence, anorexia, weight loss, nausea, vomiting or fever (Table 1). We found no significant association between assemblage A or B and clinical symptoms, in line with previous studies [29, 30]. Neither were clinical symptoms different between nitroimidazole sensitive and refractory cases (Table 2).

This study has several strengths. It is a large prospective multicentre study with a predefined treatment ladder, it includes both clinical and assemblage data, and the long inclusion period allowed for evaluation of risk of nitroimidazole failure and assemblage distribution over time. Inclusion of samples and data from a previously published Swiss treatment cohort increased the sample size for assemblage and risk factor analyses, which had not been reported in the original study [32]. There are also limitations. Parasitological confirmation of treatment response was not available in 13 patients and their response was based on clinical cure. Immunodeficient patients often have severe protracted giardiasis, but the number of patients in this group in the study was too small to reliably evaluate treatment effect. Giardiasis is a self-limiting disease in many cases, and spontaneous cure is a possibility that could have been elucidated with an untreated control group, but this was not considered ethically appropriate. Further, a single arm cohort study design was chosen rather than comparing different drug regimens due to the limited number of patients with giardiasis in our centres in a non-endemic setting.

## Conclusion

This study shows that second line combination treatment with nitroimidazole and albendazole, and third line therapy with quinacrine, are effective treatment options in nitroimidazole-refractory giardiasis. Nitroimidazole failure is an increasing problem in giardiasis, and particularly among travellers to India. Assemblage type A or B was not associated with nitroimidazole failure in this study. Further studies on nitroimidazole resistance mechanisms are needed to allow developing routine laboratory resistance testing as well as targeted antimicrobial treatment in giardiasis.

## Electronic supplementary material

Below is the link to the electronic supplementary material.

Supplementary Material 1

## Data Availability

The datasets used and analysed during the current study are available from the corresponding author on reasonable request.

## Abbreviations

PCR: Polymerase chain reaction
TID: Three times daily
SD: Single dose
BID: Two times daily
gdh: glutamate dehydrogenase gene
IQR: Interquartile range
OR: Odds ratio
CI: Confidence interval
aOR: Adjusted odds ratio
