# Supplementary material for: Nitroimidazole - albendazole combination therapy, quinacrine use, and risk factors in nitroimidazole-refractory giardiasis: a prospective multicentre observational study
Source: BMC Infect Dis. 2026 Jun 20;26:1598. doi: 10.1186/s12879-026-13833-2 (PMC13540879; doi:10.1186/s12879-026-13833-2)
Supplement: Supplementary file 1 [file 12879_2026_13833_MOESM1_ESM.docx]

***Additional file 1.***

**Supplementary Table. Amplification parameters and reaction details for real-time PCR assays**

|  | **Probe assay*** | | | | | **SYBR assay**** | | |
| --- | --- | --- | --- | --- | --- | --- | --- | --- |
| **Step** | **Temp.** | **Hold**  **(mm:ss)** | | | **No. of cycles** | **Temp.** | **Hold**  **(mm:ss)** | **No. of cycles** |
| **UNG-activation** | 52°C | 02:00 | | | 1 | NA | | |
| **Pre-incubation** | 95°C | 03:00 | | | 1 | 95°C | 03:00 | 1 |
| **Denaturation** | 95°C | 0:10 | | | 45 | 95°C | 00:10 | 41 |
| **Annealing** | 60°C | 1:00 | | |  | 64°C | 00:30 |  |
| **Elongation** | 72°C | 0:10 | | |  | 72°C | 00:02 |  |
| **Melting Curve analysis** | NA | | | | | 95°C | 00:05 | 1 |
|  |  |  |  |  |  | 65°C | 01:00 | 1 |
|  |  |  |  |  |  | 97°C | 5 acq/°C | 1 |
| **Cooling** | 40°C | | 00:30 | 1 | | 40°C | 00:30 | 1 |

*Each reaction contained a primer concentration of 450 nM, a probe concentration of 150 nM, and a 2X concentration of a Takyon No Rox Probe MasterMix dTTP Blue kit (Eurogentec, Seraing, Belgium).

**Each reaction contained a primer concentration of 250 nM, and a 2X concentration of a KAPA SYBR FAST qPCR Master Mix Kit (KapaBiosystems, Cape Town, South Africa).

In both assays it was used 2 µL template in a 25 µL reaction mix and run on a Light Cycler 480 II (Roche Diagnostics, Basel, Switzerland).
